# Prevalence and predictors of postcholecystectomy syndrome in Nepalese patients after 1 week of laparoscopic cholecystectomy: a cross-sectional study

**DOI:** 10.1038/s41598-024-55625-1

**Published:** 2024-02-28

**Authors:** Roshani Shrestha, Prangtip Chayaput, Kessiri Wongkongkam, Wallada Chanruangvanich

**Affiliations:** 1 Master of Nursing Science Program in Adult and Gerontological Nursing, Faculty of Nursing, Mahidol University, Salaya, Nakhon Pathom, 73170 Thailand; 2 Department of Nursing, Dhulikhel Hospital, Kathmandu University Hospital, Kathmandu, Nepal; 3 Department of Surgical Nursing, Faculty of Nursing, Mahidol University, Bangkok, 10700 Thailand

**Keywords:** Dyspepsia, Laparoscopic cholecystectomy, Nepal, Postcholecystectomy syndrome, Predictors, Preoperative anxiety, Prevalence, Gastroenterology, Health care, Signs and symptoms

## Abstract

Postcholecystectomy syndrome (PCS) is persistent distressing symptoms which develops following a laparoscopic cholecystectomy (LC); in cases when the condition is severe, readmission may be necessary. However, research on the prevalence of PCS and potential factors associated with PCS in Nepalese patients is still limited. An observational point-prevalence, correlational predictive cross-sectional study was conducted to determine the prevalence of PCS and examine what predicting factors including preoperative anxiety, preoperative dyspepsia, smoking, alcohol consumption, and duration of preoperative symptoms are associated with PCS. A total of 127 eligible Nepalese patients who came for follow-up after 1 week of LC at outpatient department of surgery in one single university hospital, Kathmandu, Nepal, were recruited. A set of questionnaires consisting participants' information record form, Hospital Anxiety and Depression Scale (HADS), Leeds Dyspepsia Questionnaires (LDQ), Fagerstrom Test for Nicotine Dependence (FTND), and Alcohol Use Disorder Identification Test (AUDIT) was administered for data collection. The associations between influential factors and PCS were analyzed using Binary logistic regression. 43.3% of participants reported PCS after 1 week of surgery. The findings from logistic regression analysis affirmed that the patients with preoperative anxiety (OR = 6.38, 95%CI = 2.07–19.67, p < 0.01) and moderate to severe dyspepsia (OR = 4.01, 95%CI = 1.34–12.02, p < 0.05) held the likelihood to report PCS 6.38 and 4.01 times, respectively, greater than others. The implications from study results are that screening of anxiety and patients’ tailored interventions to reduce anxiety should be implemented preoperatively. An appropriate health education about persistence of PCS and self-management should be provided to those postoperative patients.

## Introduction

Globally, cholelithiasis or gallstone is one of the foremost prevailing gastrointestinal diseases with a considerable burden on health care systems^1^. In a glance at the world, in the United States, it constitutes a significant health problem affecting 10–15% of the adult population^1,2^. In Asia, the frequency of gallstone disease was 5–10% of the population, especially among females and older population^3^. In India, the overall estimated prevalence was reported to be 2–29%^4^. In Nepal, in a multicenter ultrasonography study, the overall prevalence was found to be 4.87%^5^.

Almost 90–95% of gallstone cases develop acute cholecystitis and 10–30% of cases lead to life-threatening complications including empyema, gangrene, or perforation^6^. Cholelithiasis can lead to pancreatitis, cholangitis and hepatitis and add its complications. National Institute for Health and Care Excellence^7^ (NICE) Internal Clinical Guidelines recommend the conservative management for asymptomatic cholelithiasis and laparoscopic cholecystectomy for symptomatic cholelithiasis. Moreover, preoperative symptoms are usually used as a reference factor for the diagnosis and determination of the need for cholecystectomy^8^.

Studies from decades and more have determined that laparoscopic cholecystectomy (LC) is the gold standard treatment for symptomatic gallstone disease^7–9^. In developed countries about 90–93% of cholecystectomy was done by laparoscopic technique^9^. LC has many advantages over the standard open cholecystectomy and replaces the open technique. Some of the examples of LC include small wound size, earlier return to bowel function, shorter hospitalization, faster recovery and earlier return to normal activity, reduced pain, much less chance of postoperative scarring formation, better cosmetics and moreover, reduced overall cost^10,11^.

Postoperative results and relief of symptoms are stated after cholecystectomy^12^. But it can have some form of complications such as bile duct injury, bleeding, bile duct stricture, cholangitis, primary duct formation, injury to other organs, etc^10,13^. Moreover, sometimes cholecystectomy is unsuccessful to relieve symptoms and some patients continue to have symptoms after the surgery^14^. Persistence of symptoms had been reported since the very beginning, when cholecystectomy was started and the overall incidence after LC was found to be 13%^15^. In a recent study, the incidence of persistence of symptoms was found to be 59% in 1 week and 13% in 6 months^14^. Persistence symptoms after cholecystectomy were named postcholecystectomy syndrome (PCS).

Different form of gastrointestinal symptoms including fatty food intolerance, nausea and vomiting, heartburn, flatulence, indigestion, diarrhea, bloating or irritable bowel syndrome, mild occasional abdominal pain attacks, and severe RUQ pain with intense postcholecystectomy distress can occur after laparoscopic cholecystectomy^16–18^. The development and persistence of symptoms caused by cholecystectomy would continue for weeks to months later after cholecystectomy^19,20^. The onset of symptoms ranges from 2 days to 25 years^21^. But there remained a diagnostic and therapeutic challenge with PCS, despite the large volume of medical information regarding it^16^.

PCS is taken into consideration as a serious burden to health care systems in the United States, as 56% of patients need specific health care services for diagnosis and treatment, and it also adds sick leave and loss of productivity in the case of employed patients^18^. Among the patients with PCS, 12.5% were severe and needed hospital admission due to the severity of symptoms, and new symptoms appeared in 5–40% of cholecystectomies which affected patients’ quality of life after surgery^20^. A recent study found that 15.2% of patients were readmitted due to an additional examination for persistent abdominal pain after laparoscopic cholecystectomy^22^.

The persistence of symptoms after cholecystectomy is distressing, as even after successful surgery, patients need to be investigated for the cause of their symptoms^14^. It is even a stressful condition for those who consider that laparoscopic cholecystectomy is the best modality for the treatment of gall stone disease^23^. Some previous studies had focused on predicting factors of PCS. High trait anxiety^12,24,25^, high BMI, smoking, and alcohol consumption^26^, longer preoperative duration of symptoms, preoperative awareness, preoperative flatulence, preoperative nonspecific symptoms^27^ were considered as predicting factors of PCS. Previous studies suggested that a higher frequency of PCS is associated with age^28^, female gender^29^, longer preoperative symptoms durations, functional acalculous gallbladder, and non-inflammatory gallbladder^16,29^.

With the increase in popularity of LC, the number of patients undergoing this type of surgery has increased markedly. Nepal cannot remain away from this trend. When analyzing the 2 years’ data of a university hospital in Nepal, in the year 2018 and 2019 there were 464 and 516 cases underwent LC respectively. This data showed an increasing trend of LC for uncomplicated symptomatic cholelithiasis and cholecystolithiasis. There has been an increase in the number of cases that undergo LC in Nepal^30^. Various studies in Nepal conclude that LC is one of the safe, reliable, and promising modes that can be done successfully in a rural area in keeping the mind on clinical outcome, mortality, morbidity, and socio-economic status of the rural population^11,12,31^.

Additionally, in Nepal, the number of patients undergoing laparoscopic surgery has increased markedly with the increasing popularity of LC. Only few studies mentioned PCS but did not investigate predictive factors. PCS is underreported in Nepal, despite tons of literature regarding its being carried out in other countries. Several pieces of evidence supporting the presence of symptom persistence after LC are available to the rest of the world. There is a challenge to explore in Nepal. Since admission to discharge, the patients are less informed about the chances of having persistence of symptoms or an incident of symptoms after LC. Moreover, PCS investigation and its further management, and sufficient monitoring of patients after surgery for desirable symptomatic outcomes are required to be examined. Resources are limited in Nepal; the cost needed for such an intervention is higher.

Identification of patients with a high risk of developing persistence of symptoms after LC is very crucial in clinical nursing practice. When the LC is recommended, patients might wonder about how to relieve their symptoms and any occurrence of new symptoms after surgery^17^. Patients with PCS have several clinical symptoms, and most of them are gastrointestinal symptoms. These symptoms might neither be neglected nor inadequately addressed which could lead to severe problems and be incurable.

Most previous literature reported findings and influential factors at 1 month^14,29^, 3 months^26,32,33^ and 6 months^25,32^. Few studies noted the PCS duration within 1 week, however, most of the studies have not mentioned what happened in 1 week after surgery. We have single evidence in Nepal so far that the patients report the persistence of symptoms in 1 week, yet we still have limited evidence to support about possible predicting factors. Studying predictors of the persistence of symptoms will help clinicians identify a potential risk group and allow them to plan advanced care for them. In a developing country like Nepal, where health care resources are limited, the identification of risk groups using available predictors can help decrease the health burden. This also might reduce unnecessary care and improve the appropriate symptom outcome after LC.

### Study design

An observational point-prevalence, correlational predictive study with cross-sectional design aims to determine the prevalence of PCS and examine what predicting factors including preoperative anxiety, preoperative dyspepsia, smoking, alcohol consumption and duration of preoperative symptoms are associated with PCS in Nepalese patients following 1 week of LC. Patients who were eligible and came for follow-up after 1 week of LC at outpatient department of surgery in one single University Hospital, Nepal were enrolled. This study followed the guidelines recommended by the Strengthening the Reporting of Observational Studies in Epidemiology (STROBE).

### Participant selection and setting

All the patients, aged 18 years and above, were undergone LC and came for a follow up visit after 1 week of surgery at the surgical department of a single University Hospital, in Nepal. Sample size of 127 was calculated using G* power ^34^ analysis which is based on the predictive power of anxiety by Merten et al.^25^ A total sample of 127 patients was recruited according to inclusion criteria; (1) being undergone elective LC, (2) diagnosed with cholelithiasis, cholecystolithiasis, choledocolithiasis, post endoscopic retrograde cholangiopancreatography (ERCP) and cholecystitis, (3) having ASA grade I and II, and (4) GP-COG ^35^ equal to 9 for the age of 60 years and above. While patients who had previous abdominal surgery, carcinoma, pregnancy, and history of any psychiatric illness or taking any psychotic drugs were excluded from the study.

### Data collection and instruments

A set of questionnaires consisting participants’ information record form, Hospital Anxiety and Depression Scale (HADS)^36^, Leeds Dyspepsia Questionnaires (LDQ)^37^, and Fagerstrom Test for Nicotine Dependence (FTND)^38^ and Alcohol Use Disorder Identification Test (AUDIT)^39^ was applied for data collection. Approval from the original author has been received before using the above instruments. The translated Nepali version of HADS, FTND and GP COG was employed whereas LDQ and AUDIT were back translated by the researcher, language expert, and surgeon and nurse expert.

HADS is composed of 14 items each 7 items for anxiety sub-scale (HADS A) and depression subscale (HADS D) which is assessed separately. Each item in the scale is rated on 4 points Likert scale 0–3 where 3 indicate the maximum severity and the scores are summed^36^. The total scores range from 0 to 21 for each sub-scale and can interpret as no cases (0–7), doubtful cases (8–10), and cases (11–21). Back-translated LDQ, Nepali version, is 8 items questionnaire which consists of 2 stems for frequency and severity of dyspeptic symptoms. LDQ has a score ranging from 0 to 40 and contained questions on epigastric pain, retrosternal pain, regurgitation, nausea, vomiting, belching, early satiety, and dysphagia^37^. The frequency of the first five questions was used to determine the presence of dyspepsia and all 8 questions were required to measure a severity. For the participants who had history of smoking and alcohol consumption, FTND and AUDIT was used^38,39^.

All participants completed each questionnaire after giving an informed consent. For participants who could read Nepali, each questionnaire was self-administered; for ones who could not read, the researcher read the questionnaire out to them. This study was reported concerning the Strengthening Reporting of Observational Studies in Epidemiology (STROBE) statement^40^.

### Statistical analysis

Bivariate analyses used with a Chi-squared test and multiple logistic regression were conducted to compare two outcome groups (cases vs non-cases of PCS) and identify the associated factors for PCS. The variables for predicting factors including preoperative anxiety, preoperative dyspepsia, smoking, alcohol consumption, and duration of preoperative symptoms were analyzed in relation to the occurrence of PCS. The Hosmer Lemeshow and the Nagelkerke R^2^ tests were reported for the logistic regression analysis. SPSS statistics version 25.0 for Windows (IBM Corp., Armonk, NY, USA) was utilized for statistical analysis of all data.

### Ethical considerations

After the ethical approval from Institutional Review Board of Faculty of Nursing (NS-IRB) (COA No. IRB-NS2021/599.0802), Mahidol University, Thailand, and Institutional Review Board of Kathmandu University, School of Medical Sciences, Dhulikhel Hospital (IRC KUSMS Approval No 28/2021), Nepal, a data collection was started. Written informed consent was obtained from each participant before enrolling in the study.

## Results

### Characteristics of participants

As Table 1, this study showed that 43.3% of participants had PCS after 1 week of LC. Large proportions of participants were female (72.4%). Nearly half of participants (48%) aged 18–40 with mean age of 42.88 ± 13.12 years. Almost half of the female participants (46.7%) had PCS after 1 week of LC. Likewise, more than half of the participants (54.5%) aged more than 60 developed PCS. The mean age of participants who had PCS was 44.93 ± 13.81 years.

**Table 1:** Frequency, percentage, mean, and standard deviation of participant characteristics and PCS (n = 127).

Characteristics	Total n (%)	PCS	
		Yes (n = 55)	No (n = 72)
Gender			
Female	92 (72.4)	43 (46.7)	49 (53.3)
Male	35 (27.6)	12 (34.3)	23 (65.7)
Age (years)			
18–40	61 (48.0)	25 (41.0)	36 (59.0)
41–60	55 (43.3)	24 (43.6)	31 (56.4)
> 60	11 (8.7)	6 (54.5)	5 (45.5)
Mean ± SD (years)	42.88 ± 13.12	44.93 ± 13.81	41.32 ± 12.44
Underlying diseases			
Yes	50 (39.4)	29 (58.0)	21 (42.0)
Hypertension	26 (52.0)	15 (57.7)	11 (42.3)
Diabetes mellitus	15 (30.0)	11 (73.3)	4 (26.7)
Hypothyroidism	9 (18.0)	3 (33.3)	6 (66.7)
Cardiovascular disease	3 (6.0)	2 (66.7)	1 (33.3)
Others	6 (12.0)	4 (66.7)	2 (33.3)
No	77 (60.6)	26 (33.8)	51 (66.2)

Out of 127 participants, 39.4% had one or more than one underlying disease. Among them, half the participants (52%) had hypertension. Secondly, one–third of the participants (30%) had Diabetes Mellitus, and only 6% had cardiovascular diseases. Moreover, 73.3% of participants who had Diabetes Mellitus developed PCS to the same degree as 66.7% of participants with cardiovascular disease and other diseases.

As Table 2, preoperative anxiety and depression were assessed using HADS. The mean preoperative anxiety score was 7.08 ± 4.34. The participants who scored 11–21 (case) were 18.9%. However, a larger proportion of participants (79.2%) who scored 11 or more experienced PCS.

**Table 2:** Frequency, percentage and standard deviation of preoperative anxiety using HADS and preoperative dyspepsia, and PCS (n = 127).

Variables	Total n (%)	PCS	
		Yes (n = 55)	No (n = 72)
Preoperative anxiety			
0–7 (No case)	82 (64.6)	27 (32.9)	55 (67.1)
8–10 (Doubtful case)	21 (16.5)	9 (42.9)	12 (57.1)
11–21 (Case)	24 (18.9)	19 (79.2)	5 (20.8)
Mean ± SD	7.08 ± 4.34	8.94 ± 4.81	5.66 ± 3.33
Preoperative dyspepsia			
0 (No dyspepsia)	36 (28.3)	9 (25.0)	27 (75.0)
1–8 (Very mild to mild)	53 (41.7)	22 (41.5)	31 (58.5)
9–40 (Moderate to Severe)	38 (30.0)	24 (63.2)	14 (36.8)
Mean ± SD	6.34 ± 6.37	8.89 ± 7.51	4.40 ± 4.50

Preoperative dyspepsia was assessed by the Leeds Dyspepsia Questionnaire. The mean preoperative dyspepsia score was 6.34 ± 6.37. The result showed that 41.7% of participants had very mild to mild dyspepsia, followed by 30% of participants had moderate to severe dyspepsia, and 28.3% of participants had no preoperative dyspepsia. More than half of the participants (63.2%) with moderate to severe dyspepsia had PCS, followed by 41.5% of participants with very mild to mild dyspepsia.

In this study, majority of participants (77.2%) were non- smoker, whereas 22.8% were smoker (n = 22). The result presented that a proportion of participants who had PCS and ones who had no PCS was almost same. Likewise, third-fourth of the participants (75.6%) did not consume alcohol. Almost equal proportion of participants who consumed alcohol and did not consume alcohol had PCS as 41.9% and 43.8%, respectively. This present study pointed out that the mean preoperative symptom duration was 16.64 ± 27.30 months. Nearly half of the participants (48.5%) having symptoms duration more or equal to 12 months experienced PCS.

### Associations between the studied predicting factors and PCS

Results from Chi-square analysis reported that only two studied variables were statistical significantly associated with PCS in this study. These factors include preoperative anxiety (χ^2^ = 16.17, p < 0.001), and preoperative dyspepsia (χ^2^ = 11.08, p < 0.01). Participants, who were cases, were more likely to develop PCS (79.2%). Similarly, more than half of participants with moderate to severe dyspepsia were more likely to have PCS (63.2%). The results of Chi-square test is shown in Table 3.

**Table 3:** Associations between preoperative anxiety, preoperative dyspepsia, smoking status, alcohol consumption, preoperative symptoms duration, and PCS (Chi-square test, n = 127).

Variables	PCS		χ^2^	p-value
	No (n = 72)	Yes (n = 55)		
Preoperative anxiety			16.17	< 0.001
0–7 (No case)	55 (67.1)	27 (32.9)		
8–10 (Doubtful case)	12 (57.1)	9 (42.9)		
11–21 (Case)	5 (20.8)	19 (79.2)		
Preoperative dyspepsia			11.08	< 0.01
0 (No Dyspepsia)	27 (75.0)	9 (25.0)		
1–8 (Very mild to mild)	31 (58.5)	22 (41.5)		
9–40 (Moderate to severe)	14 (36.8)	24 (63.2)		
Smoking status			0.38	0.54
No/Never	57 (58.2)	41 (41.8)		
Yes	15 (51.7)	14 (48.3)		
Alcohol consumption			0.31	0.86
No/Never	54 (56.3)	42 (43.8)		
Yes	18 (58.1)	13 (41.9)		

As Table 4, to identify the predictors of the PCS among patients after 1 week of LC in a university hospital in Nepal, a binary logistic regression test was employed. The study findings revealed that having anxiety preoperatively (Cases) (OR = 6.38, 95%CI = 2.07–19.67, p < 0.01) and having moderate to severe dyspepsia (OR = 4.01, 95%CI = 1.34–12.02, p < 0.05) were more likely to experience PCS.

**Table 4:** Logistic regression analysis of preoperative anxiety, preoperative dyspepsia, smoking, alcohol consumption, preoperative symptoms duration, and PCS (n = 127).

Variables	B	S.E	Wald	OR Exp (B)	95%CI	p-value
Preoperative anxiety						
0–7 (No case)	Ref					
8–10 (Doubtful case)	0.20	0.52	0.15	1.23	0.44–3.42	0.697
11–21 (Case)	1.85	0.57	10.42	6.38	2.07–19.67	< 0.01
Preoperative dyspepsia						
No dyspepsia	Ref					
Very mild to moderate	0.72	0.50	2.01	2.05	0.76–5.50	0.156
Moderate to severe	1.39	0.56	6.15	4.01	1.34–12.02	< 0.05
Smoking status						
No/never	Ref					
Yes	0.29	0.50	0.34	1.34	0.49–3.60	0.562
Alcohol consumption						
No/never	Ref					
Yes	− 0.27	0.50	0.29	0.76	0.29–2.03	0.592
Preoperative symptom duration						
< 12 months	Ref					
≥ 12 months	0.92	0.46	0.04	1.10	0.45–2.68	0.839

Note: Hosmer and Lemeshow test: χ^2^ = 2.59, df = 6, p-value = 0.86, Cox & Snell *R*^2^ = 0.17, Nagelkerke *R*^2^ = 0.23, Predictive correct = 70.1

## Discussion

In this present study, the PCS was determined by the attending surgeon at the time of follow-up after 1 week of LC according to the definition of PCS, history taking, and physical examination. The prevalence of PCS in this study was 43.3% which is higher than other previous studies^26,41,42^. Nepalese participants who had PCS were asked for follow-up after one month in the outpatient department, and whether symptoms persisted, they would be taken for another treatment plan.

The finding of this current study was similar to Angeline and Lalisang’s study that noted the prevalence of PCS in Indonesia to be 45.5%, but in the different periods from soon after surgery to 18 months after surgery^27^. According to the study of Kouloura et al.^21^, the onset of symptoms in PCS occurred from 2 days to 25 years after LC. Only a few studies had reported the prevalence of PCS after 1 week of cholecystectomy^15^. Otherwise, the prevalence of PCS in our study was quite smaller than Arora et al.^14^ which reported the PCS of 58% after 1 week, 39.1% in 1 month, 23.2% in 3 months, and 13% in 6 months. The other study reported a lower prevalence of 17.1% when followed up after one month of LC^41^. Another study found that 23% of study participants had persisted preoperative symptoms after 4 months of surgery^26^. Likewise, a study reported the prevalence to be 16.66% after 3–10 months after surgery^42^. Similar to several reports, another study noted 19.8% of PCS where the exact follow up period after surgery was not mentioned^43^.

Most of those aforementioned studies had a lower rate of PCS when compared to the present study. As we discussed the prevalence of PCS was higher when followed up after 1-week whereas in other studies where the followed-up period was longer. In a study, they reported decreasing prevalence of PCS as an increase in time duration^14^. This might be the reason why the current study had a higher rate of PCS as participants were followed up only after 1 week of surgery. Also, due to the alteration of bile flow after the removal of the gallbladder, it resulted in various gastrointestinal symptoms like bloating, belching, flatulence, etc. The body needed to adopt the physiological changes and patients might have those symptoms during adaptation^7,16^. This might be another reason why this study had a higher prevalence of PCS.

Corresponding with the previous study reported 38.75% of patients had preoperative anxiety that underwent LC. A main reason for preoperative anxiety was found to be fear of post-operative pain and of anesthesia^44^. In this current study, 18.9% of participants had preoperative anxiety. This result was in line with the previous study reported high trait anxiety as the only predictor of persistence of biliary symptoms 6 weeks after cholecystectomy^12^. However, there was a difference in measurement tools to measure the preoperative anxiety in a previous study and current study, the results were congruent. The previous study reported there was the presence of some sort of anxiety before surgery and how psychological distress added to the pathophysiology of PCS. Therefore, the result of this study that preoperative anxiety as a predictor of PCS could be explained.

Regarding the reviewed literatures, there were no recent researches that studied preoperative dyspepsia as a predicting factor of PCS. However, this result can be in line with a decade-old^25^ where the researcher proposed preoperative reports of dyspeptic symptoms to predict new dyspeptic symptoms after 6 months. Even though the measurement of preoperative dyspepsia and time duration when the patients were followed up were different from current study. However, our results were similar in that preoperative dyspepsia could predict the PCS.

This result could be supported insight into the pathophysiology of PCS and dyspepsia. There were various causes of dyspepsia including alteration of gastric motility, visceral hypersensitivity, *H. pylori* infection, psychological factors, etc^45^. An absence of gall bladder after surgery results in a continuous drainage of hepatic bile to the duodenum which causes rapid enterohepatic bile cycling and surplus passage of bile juice into the duodenum^16^. This causes an increase in duodenal gastric reflux and leads to the sphincter of Oddi incompetence which further added alteration of gastric motility^16,29^. Moreover, the bile duct needed 1–2 weeks to recover from dilatation after surgery. This might be a reason why patients, who had preoperative dyspepsia, might develop new symptoms of dyspepsia after surgery^6,18^.

Additionally, preoperative dyspepsia is one of the significant predictors for PCS. Nurses should preoperatively screen the patients who have a treatment plan of LC for its dyspepsia level of severity and manage properly. An individual-tailored patient’s education on how to manage dyspepsia and persistent dyspeptic symptoms after surgery should be intervened. As a preoperative anxiety was one of the statistically significant predictors of PCS, evaluating all patients who were planned for LC and finding out its cause will help managing occurred anxiety suitably before getting an operation. An appropriate health education about possibility of having persistent PCS should be explained to preoperative patients with LC, and what self-management of symptoms should be followed.

## Conclusions

To summarize, this study revealed that nearly half of patients with LC reported PCS. Postoperative female patients whose age greater than 60 years and having underlying disease are more likely to suffer PCS than other groups. The preoperative anxiety and preoperative dyspepsia were significant predictors of PCS. The patients with preoperative anxiety and moderate to severe dyspepsia held the likelihood to experience PCS 6.38 and 4.01 times, respectively, greater than others. Based on these results, screening and prompt management of preoperative anxiety and dyspepsia as to minimize risk for PCS along with specific patient's education regarding PCS information in patients planned for LC should be initiated. A proper postoperative follow-up and monitoring of PCS should be provided accordingly.

### Strength of the study

Since it is limited data of PCS and its predictors in Nepali context, this study revealed the prevalence of PCS after 1 week of LC is 43.3% which can be used as the base for further study. Results of this study can be used to identify a risk group and provide appropriate tailored education as to manage existing symptoms. This study also indicated an importance of monitoring the patients following LC for symptomatic outcomes at follow up time.

### Limitation of the study

The diagnosis of PCS is only preliminary diagnosis, yet it would not be definitive. This study was conducted in a single setting, university hospital, in Nepal, and only included patients who came for 1 week follow-up, thus the results may not be generalized. A nature of cross-sectional study design would not acknowledge to measure changes over time condition, which was impractical to infer causality and comparison of interest outcomes. The repeated measure case–control study would be a challenge for a future research.

## Data Availability

According to the regulation of the Nepal, data will be limited to provide outside the Nepal. However, the data that support the findings of this study will be available from the principal investigator, Roshani Shrestha (roshani88.sth@gmail.com) upon reasonable request.
